# Supplementary material for: The causal relationship between the human gut microbiota and pyogenic arthritis: a Mendelian randomization study
Source: Front Cell Infect Microbiol. 2024 Nov 26;14:1452480. doi: 10.3389/fcimb.2024.1452480 (PMC11629706; doi:10.3389/fcimb.2024.1452480)
Supplement: Supplementary file 1 [file DataSheet1.zip › mendelian test/ebi-a-GCST90027495.csv_leave_one_out.pdf]

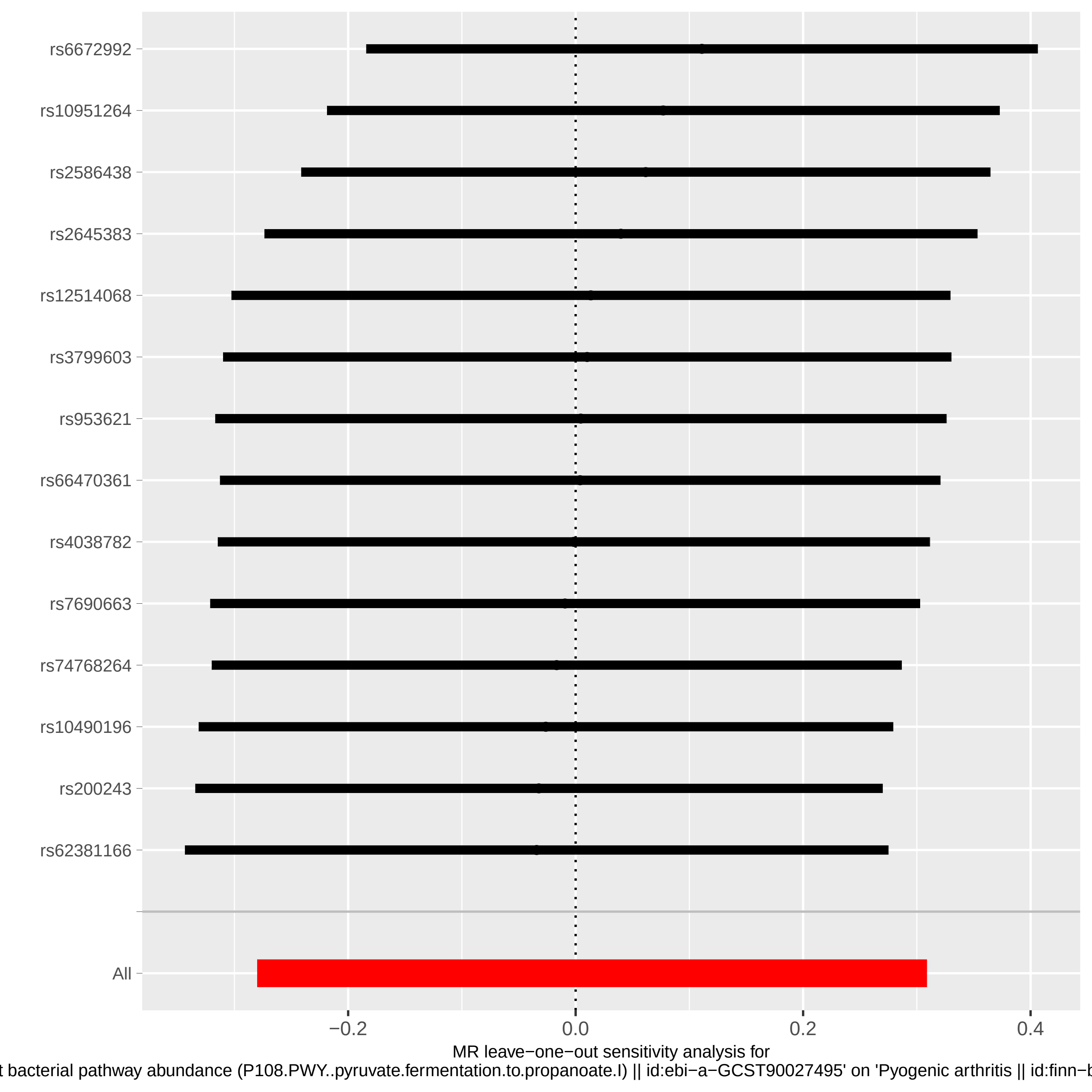

rs6672992
rs10951264
rs2586438
rs2645383
rs12514068
rs3799603
rs953621
rs66470361
rs4038782
rs7690663
rs74768264
rs10490196
rs200243
rs62381166
All
−0.2
0.0
0.2
0.4
MR leave−one−out sensitivity analysis for
t bacterial pathway abundance (P108.PWY..pyruvate.fermentation.to.propanoate.I) || id:ebi−a−GCST90027495' on 'Pyogenic arthritis || id:finn−b
